# Microstructural and Mechanical Characterization of Newly Developed Zn-Mg-CaO Composite

**DOI:** 10.3390/ma15238703

**Published:** 2022-12-06

**Authors:** Jan Pinc, Jiří Kubásek, Jan Drahokoupil, Jaroslav Čapek, Dalibor Vojtěch, Andrea Školáková

**Affiliations:** 1 Institute of Physics of the Czech Academy of Sciences, Na Slovance 1999/2, 182 21 Prague, Czech Republic; 2 Department of Metals and Corrosion Engineering, Faculty of Chemical Technology, University of Chemistry and Technology, Technická 5, Praha 6—Dejvice, 166 28 Prague, Czech Republic

**Keywords:** extrusion, biodegradable metals, zinc, EBSD, compressive properties, ball milling, powder metallurgy, µCT

## Abstract

In this study, the Zn-0.8Mg-0.28CaO wt.% composite was successfully prepared using different conditions of ball milling (rotations and time) followed by a direct extrusion process. These materials were characterized from the point of view of microstructure and compressive properties, and the correlation between those characteristics was found. Microstructures of individual materials possessed differences in grain size, where the grain size decreased with the intensified conditions (milling speed and time). However, the mutual relation between grain size and compressive strength was not linear. This was caused by the effect of other factors, such as texture, intermetallic phases, and pores. Material texture affects the mechanical properties by a different activity ratio between basal and pyramidal <c + a> slips. The properties of intermetallic particles and pores were determined in material volume using micro-computed tomography (µCT), enhancing the precision of our assumptions compared with commonly applied methods. Based on that, and the analysis after the compressive tests, we were able to determine the influence of aspect ratio, feret diameters, and volume content of intermetallic phases and pores on mechanical behavior. The influence of the aspects on mechanical behavior is described and discussed.

## 1. Introduction

Zinc and its alloys have been considered promising candidates for the fabrication of biodegradable implants for over 10 years [1,2,3,4]. During this period, a significant amount of advanced knowledge concerning the influence of alloying elements [5,6,7], mechanical behavior [7,8,9], degradation process [10,11] and interaction between the implant and the tissues [12,13] was obtained and published. Based on these publications, the main advantages and disadvantages of zinc-based materials for medical use can be derived and utilized. The most substantial benefit of zinc is hidden in the degradation process under physiological conditions [14]. The degradation rate of pure zinc is almost ideal, but in most of the cases, zinc-based alloys are preferred over pure metal (e.g., ZnMgCa [15], ZnMgSr [16], ZnMnLi [5,17]) due to the resulting mechanical performance of the implant. Besides the mechanical properties, the alloying elements added into zinc also affect the corrosion behavior by the activity of micro-galvanic cells between present intermetallic phases and the Zn matrix [18,19]. This often leads to a predominant dissolution of one alloy component and subsequent localized corrosion attack. In addition to the localized corrosion attack, relatively poor adhesion of the cells to the material surface is the main disadvantage [20]. However, even alloying cannot lead to the mitigation of all mechanical drawbacks. To solve such a complex problem, applying suitable processing routes leading to microstructure refinement and a change in the material texture is necessary [21,22]. Such procedures allow us to increase mechanical performance and modify them in individual directions. This could be beneficial for concepts of the design of the material mimicking bone. To reach similar values of mechanical characteristics as in the case of bone, it is also necessary to know the phenomena occurring during the material deformation to modify or tune all the material properties.

As was mentioned in the previous paragraph, the presence of alloying elements should enhance the mechanical properties and biological interaction of the material with the surrounding tissue. The biodegradable zinc-based alloys are often alloyed by low amounts of micro and macro-essential elements such as Mg [23], Ca [6], Sr [13], Li [24], and Mn [25]. The addition of such elements leads, in most cases, to the formation of intermetallic phases (CaZn_13_, SrZn_13_, Mg_2_Zn_11_, LiZn_4_, MnZn_13_) strengthening the material. It was shown in several publications that the presence of these intermetallic phases is the main factor leading to an increase in the mechanical performance of the obtained material. The differences in strength between zinc and Zn-X binary alloys (X = Mg, Ca, Sr) observed by Li et al. [6] can be used as evidence. The addition of these elements and the presence of the intermetallic phases lead to the enhancement of strength in the order of hundreds of percent. More precisely, the addition of the elements increased the strength by 140–160 MPa. Besides these elements, more complex Ca-based compounds are often added to biodegradable alloys [26,27,28] to enhance biocompatibility and obtain a composition more similar to bone [29]. However, the intermetallic phases and the complex compounds could act as stress concentrators which can deteriorate the ductility of the material [30]. To minimize such effects, the materials are often processed using thermomechanical treatment. The treatment often increases strength through the refinement of the intermetallic phases and leads to the refinement of the Zn-based matrix [16].

Extrusion is a widely used method for the thermomechanical processing of metallic materials [31,32,33]. The process is relatively simple and often leads to microstructure refinement, minimizing defects, and creating mechanical anisotropy in individual directions [34,35]. The refinement of the microstructure is often achieved by dynamic recrystallization, which is affected by the temperature and stored strain during the process [36]. The elevated temperature during the processing is essential, especially in the case of zinc. The reason is not primarily connected with the recrystallization process, but directly with the processing itself, which is significantly limited at ambient temperature due to the hcp crystal structure of zinc. Therefore, zinc is often processed at elevated temperatures to ensure material production without significant material damage. The aforementioned anisotropy of mechanical performance is caused by the rearrangement of the microstructure in the direction of the extrusion [37]. The material loaded in the direction of the extrusion often exhibits better mechanical performance in contrast with that loaded in the direction perpendicular to the extrusion [16]. In addition, the process often leads to the formation of deformation texture affecting the prevailing deformation mechanism and thus the mechanical properties [38].

In this study, we successfully prepared a novel Zn-0.8Mg-0.28CaO (wt.%) alloy using powder metallurgy techniques (high energy milling, extrusion). The composition was chosen according to our previous studies concerning ZnMgCa alloys [15] (the same content of Mg and Ca). In addition, the microstructure and compressive behavior were characterized, and mutual correlations between individual aspects were found. According to the best of our knowledge, the CaO compound has not been used in this way by other authors for the preparation of biodegradable metallic composites.

## 2. Materials and Methods

The powder of pure zinc (Sigma-Aldrich—Praha, Czech Republic, 20–30 mesh, 99.8 wt.%), magnesium (AlfaAesar—Kandel, Germany, −100 + 200 mesh, 99.6 wt.%), and calcium oxide (Penta—Katovice, Czech Republic, p.a., non-defined particles size) were mixed to reach the desired composition (Zn-0.8Mg-0.28CaO; wt.%). A total of 35 g of the prepared mixture was milled at ambient temperature with an addition of 0.5 g of stearic acid (PCA–process control agent) in ZrO_2_ milling jars using a high-energy ball mill Emax Retsch. ZrO_2_ milling balls with 10 mm in diameter were used for the process. To minimize the influence of the environment, the processes connected with the mixture manipulation were performed in the glovebox under an argon atmosphere (filling of the milling jar, pressing, etc.). The details of preparation of powder mixture are described in our previous work [39]. The conditions of the milling process are summarized in Table 1.

The milled mixtures were pressed into tablets of 20 mm in diameter using a universal testing machine LabTest 5.250SP1-VM (LABORTECH s.r.o., Opava, Czech Republic). The force of 80 kN with a dwell of 5 min was used for the pressing. The green bodies prepared in this way were extruded at 300 °C ± 1.5 °C using a customized hydraulic press. The extrusion ratio was set to be 10:1. As a lubricant, a high-temperature Gleit-µ HP 505 paste was used. Extruded rods were quenched into water immediately after the process to hinder the post-dynamic recrystallization.

**Figure 1:**
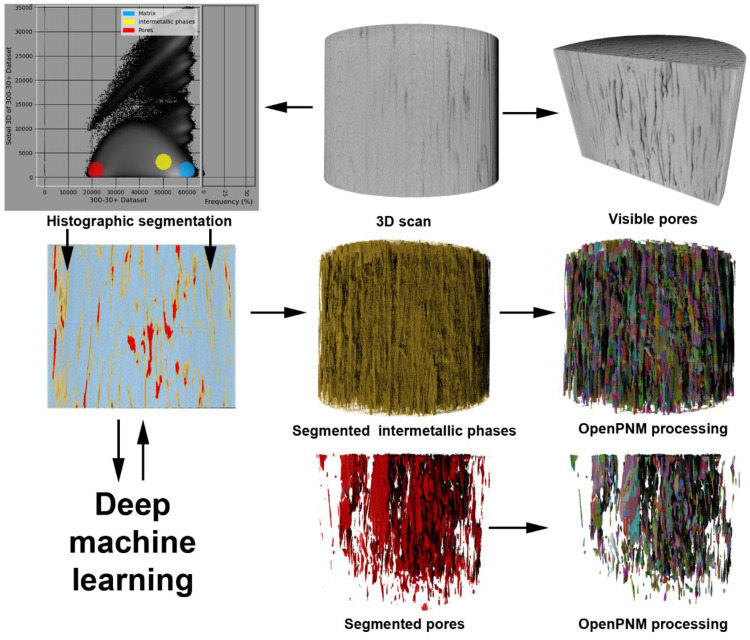
Scheme of the intermetallic particles and pores characterization.

After extrusion, the end parts of the rods were cut in order to restrict the inhomogeneities in the structure of the materials. Subsequently, five samples were cut from the center of the rod and used for microstructure characterization (1×) and compressive testing (4×). The samples for the microstructure observation were cut in half by an electro-erosive machine to obtain samples with parallel sides. Such prepared samples were subsequently analyzed using SEM. A few rows of intermetallic phases and zinc matrix were present in the observed area to determine the influence of particles on the closest surrounding of the Zn matrix. In addition, the analyses were done approximately in the middle of the sample to avoid the side areas affected by friction. Samples used for the characterization of the microstructure were ground using SiC sandpapers (up to P4000) and electrochemically polished in the solution of ethanol and phosphoric acid in a 1:1 volume ratio. The conditions of the polishing were: 10 min, 4 V, −15 °C ± 1.5 °C, and stainless steel was used as a counter electrode. After polishing, the samples were rinsed with distilled water and ethanol to remove all phosphates from the material surface. The microstructure was characterized using an FEI 3D Quanta 3D field-emission-gun DualBeam scanning electron microscope equipped with an EDS detector with EDAX Genesis software v.5.2. and electron backscattered diffraction (EBSD) detector TSL/EDAX Hikari. Acquired EBSD data were processed using TSL OIM8 software v. 8.0. The prepared samples were also characterized using micro-computed tomography Zeiss (µCT) at ambient temperature and the collected data were processed using the Dragonfly software v. 2022.1 (Object Research Systems (ORS) Inc, Montreal, Canada, 2020; software available at http://www.theobjects.com/dragonfly, accessed on 13 November 2022). Individual material phases were distinguished using histographic segmentation. During this segmentation, the pores were characterized by dark pixels with intensities in the left arc of constructed 3D histogram. On the contrary, the Zn matrix was represented by bright pixels (the right arc of the histogram). This specification was confirmed by observation of 2D and 3D slices. Subsequently, the segmented scans were processed by a deep learning tool (semantic segmentation, n = 3) with 21 million parameters counted. The previously mentioned procedure was applied to the sample 300 RPM_30 min. Afterwards, the model was applied to the rest of the prepared materials. Individual phases, formed by the segmentation process, were analyzed using the open pore network model (OpenPNM) [40] tool to determine properties such as aspect ratios, maximal feret diameters, volume/surface, or content of pores and intermetallic phases. The resolution of the µCT reached 6.6 µm/pixel. It should be mentioned that the resolution can lead to a slightly higher error value in the case of 700 RPM_60 min due to the dense distribution of the thin rows of intermetallic phases. The process of µCT characterization is schematically shown in Figure 1.

Compressive properties were measured in normal (ND) and extrusion (ED) directions using Instron 5882 machine at ambient temperature. Cuboid samples with a side length of 3 mm and height of 4.5 mm were cut from the extruded rod using an electro-erosive machine Agiecharmilles FI240 (EEC) and compressed with a ram speed of 0.27 mm·min^−1^. Three samples were always used for the measurements and the tests were stopped manually in ND due to the absence of ultimate compressive strength. The values of compressive yield strength (CYS) and ultimate compressive strength (UCS) were evaluated as compressive stress at 0.2% of plastic deformation and as the maximum stress, respectively. Strain hardening (n) and strength (K) coefficients were determined from the linear approximation of the true stress–strain curves in logarithmic scale at appropriate intervals.

## 3. Results

### 3.1. Microstructure

**Figure 2:**
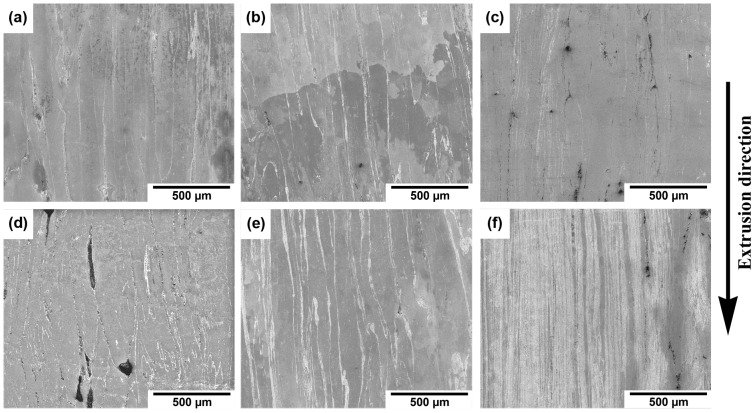
Microstructure of as-extruded (**a**) 300 RPM_30 min; (**b**) 500 RPM_30 min; (**c**) 700 RPM_30 min; (**d**) 300 RPM_60 min; (**e**) 500 RPM_60 min, and (**f**) 700 RPM_60 min alloys.

The microstructure of prepared materials was, in the initial stages of characterization, observed using SEM, and the microstructures of individual materials are shown in Figure 2. At first sight, the materials consisted of three components: zinc matrix, rows of intermetallic phases, and pores. Differences between individual samples were related to the character and content of pores and the intermetallic phases. These differences will be described in detail in the text concerning the intermetallic particles. As can be seen from Figure 2c,f, the increase in milling time and rotation speed led to the denser arrangement of the intermetallic phases. The only exception was the group of samples milled at 300 RPM, where the interrow distance was similar at both milling times. The composition of the intermetallic phases and their quantitative analysis were already published in our previous publication concerning these materials [39]. Briefly, the presence of Mg_2_Zn_11_, MgZn_2_, and CaZn_13_ phases was found in the materials with changes in mutual ratios. In addition, it was found that the presence of these intermetallic phases was strictly localized to the surface of the milled Zn particles, suggesting their presence on the interface between individual particles. Last but not least, the MgZn_2_ phase gradually transformed into Mg_2_Zn_11_ with the increase in milling energy.

**Figure 3:**
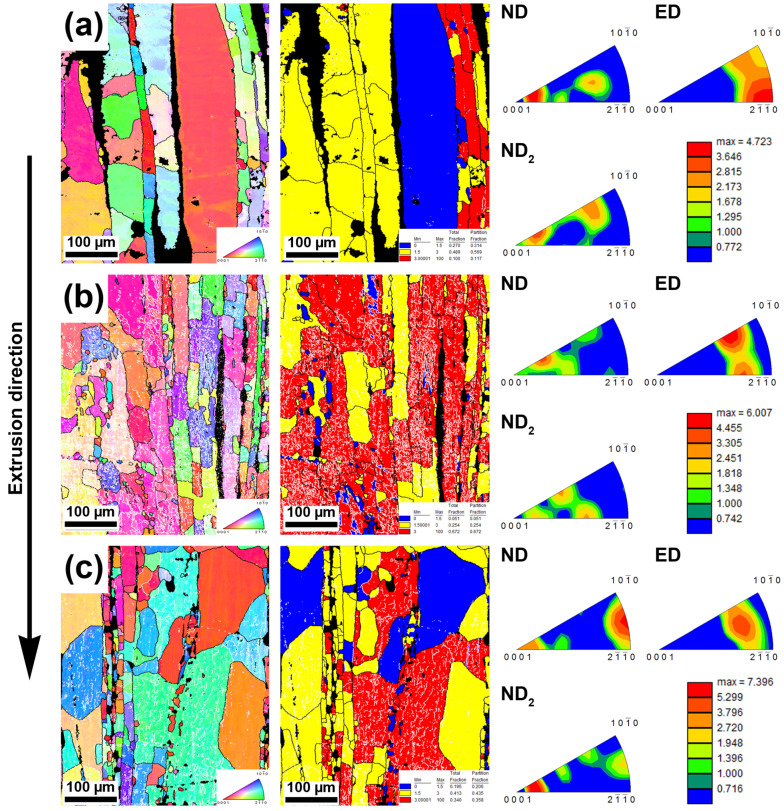
IPF map, GOS map, and IPFs of samples milled for 30 min at (**a**) 300 RPM, (**b**) 500 RPM, and (**c**) 700 RPM and subsequently extruded at 300 °C; black lines–high angle boundaries; white lines–low angle boundaries; black colored areas in IPF maps represent non-indexed intermetallic regions.

An inverse pole figure map (IPFm), grain orientation spread (GOS), and inverse pole figures (IPFs) of the samples milled for 30 min are shown in Figure 3. The differences in grain size, the content of low angle boundaries (LAGBs), and the ratio of recrystallized grains and texture were observed between individual materials. The average value of the grain size reached 30 µm in the case of a 300 RPM_30 min sample with the presence of larger grains (>70 µm) representing the majority of the analyzed area. In addition, a low amount of LAGBs were present in the microstructure. Recrystallized (blue) and transition (yellow) grains made up 27% and 49% of the area, respectively. The texture of the material milled at the minimal energy (300 RPM) was close to the common texture of extruded metals with hcp crystal structure (c/a ≥ Mg). Basal planes were aligned about 9°–25° to ND, while the crystallographic directions were tilted by 1°–16° from the right arc of the orientation triangle in ED. With the gradual increase in rotation speed to 500 and 700 RPM, a decrease in grain size (15 µm) was observed. Interestingly, the average grain size was not changed between 500 and 700 RPM (number fraction); however, the number of LAGBs decreased with an increase in rotation speed. Moreover, the portion of recrystallized and non-recrystallized grains (red) significantly changed. The presence of non-recrystallized grains had the same trend as in the case of LAGBs. More precisely, the content of non-recrystallized grains decreased from 67% (500 RPM_30 min) to 34% (700 RPM_30 min). As can be seen from the inverse pole figures, the occurrence of crystallographic directions close to the right arc of the orientation triangle increased in ND with rotations. In addition, the highest texture index was observed in the case of the sample milled at 700 RPM.

**Figure 4:**
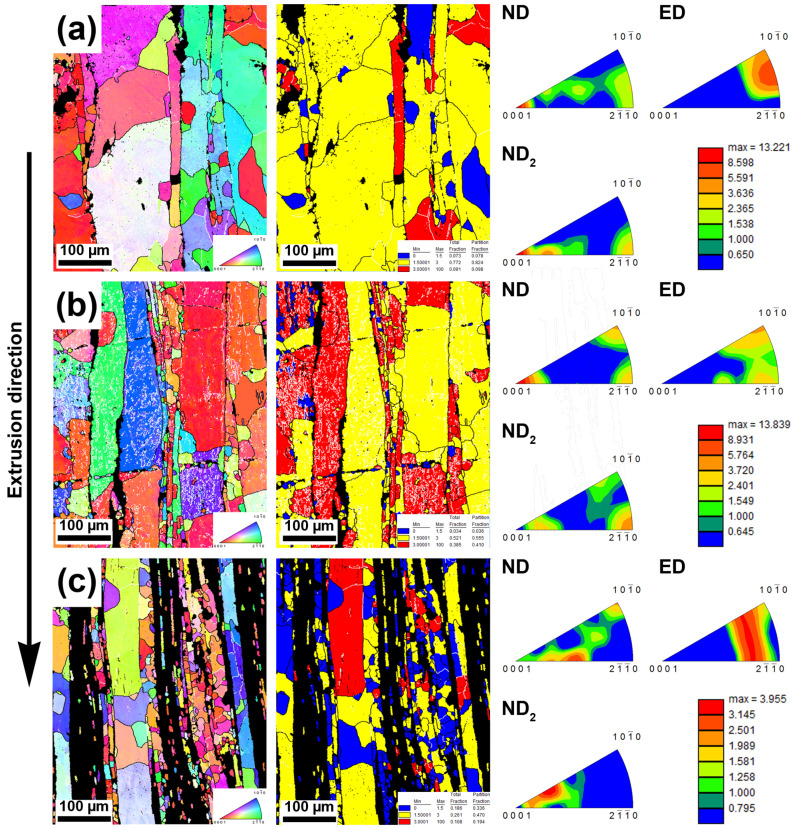
IPF maps, GOS map, and IPFs of the samples milled for 60 min at (**a**) 300 RPM, (**b**) 500 RPM, and (**c**) 700 RPM and subsequently extruded at 300 °C; black lines–high angle boundaries; white lines–low angle boundaries; black colored areas in IPF maps represent non-indexed intermetallic regions.

The IPF maps, GOS, and IPF were calculated also for the samples milled for longer times (60 min), and the results are shown in Figure 4. Grain size of the 300 RPM_60 min sample reached 25 µm with the presence of a large number of grains up to 20 µm, which is a value lower by approximately 33% compared with the 300 RPM_ 30 min sample. In addition, the microstructure contained the majority of the transition grains (82%) and 18% of recrystallized and non-recrystallized grains with equal percentual distribution between them. Furthermore, a relatively strong texture with the predominant occurrence of basal planes in ND was found. As well as in the case of 500 RPM_30 min, the sample 500 RPM_60 min possessed a large content of LAGBs and an average grain size of 16 µm. Moreover, the amount of non-recrystallized grains increased from 8% (300 RPM_60 min) to 41% (500 RPM_60 min). Interestingly, the texture was concentrated on all edges of the orientation triangle without significant deflections from these corners in ND. However, most of the planes were basal in ND, which is in good agreement with texture in ED (Figure 4b). At first sight, a high amount of non-indexed points is visible in Figure 4c. These points correspond to intermetallic phases, which are predominantly etched during polishing as a consequence of the activity of micro-galvanic cells. In addition, the microstructure of 700 RPM_60 min possessed, when compared with other samples, a relatively fine grain structure with a visible increase in fully recrystallized grains ratio (34%). From the texture point of view, the basal planes were tilted approximately by 30° in ND and the strength of the texture was the lowest of all analyzed samples, even in comparison to 30 min samples.

**Figure 5:**
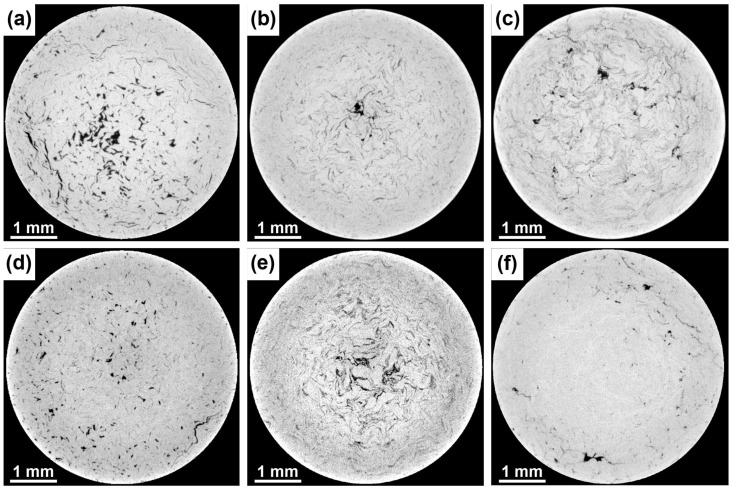
The distribution of intermetallic phases in 2D slices of the (**a**) 300 RPM_30 min, (**b**) 500 RPM_30 min, (**c**) 700 RPM_30 min, (**d**) 300 RPM_60 min, (**e**) 500 RPM_60 min, and (**f**) 700 RPM_60 min samples in the ED obtained by µCT; black and dark grey areas represent intermetallic particles.

**Figure 6:**
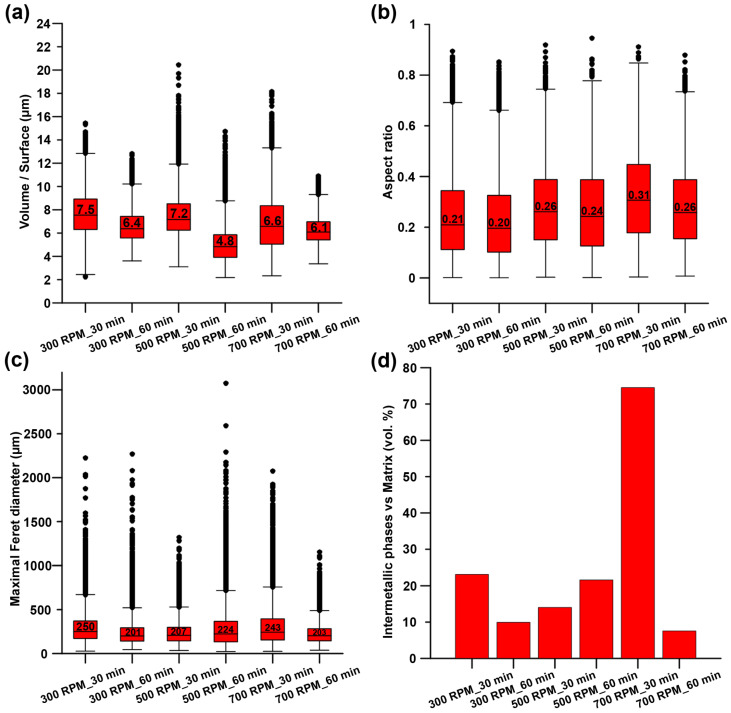
Graphs representing the (**a**) volume/surface, (**b**) aspect ratio, (**c**) maximal Feret diameter, and (**d**) content of the intermetallic phases (vol.%) in the materials prepared under different conditions.

**Figure 7:**
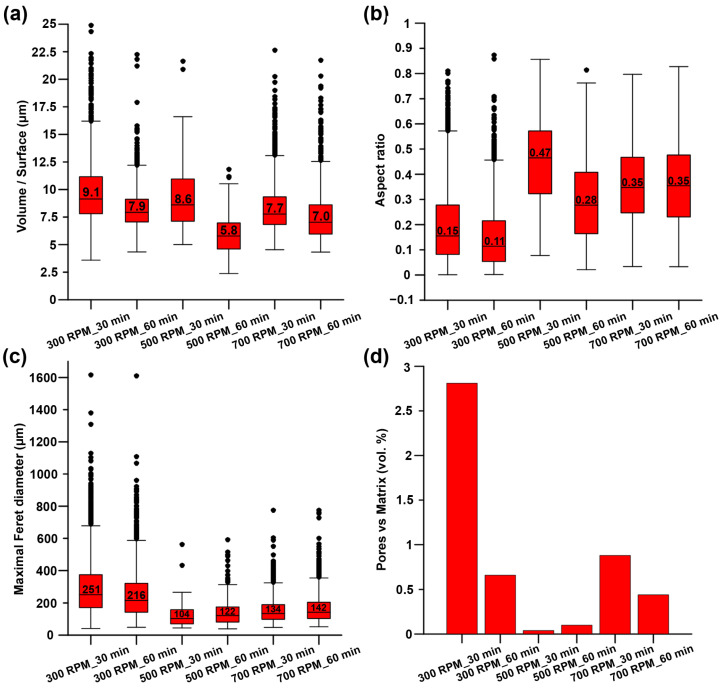
Graphs representing the (**a**) volume/surface, (**b**) aspect ratio, (**c**) maximal Feret diameter, and (**d**) content of the pores (vol.%) in the materials processed under different conditions.

The distribution of the intermetallic phases is an important factor affecting the mechanical properties of materials. However, there is a problem with the determination of the distribution via commonly used metallographic methods. Therefore, the volume characterization of the intermetallic phases and the pores was done using µCT and the results are shown in Figure 5, Figure 6 and Figure 7. As can be seen in Figure 5, the distribution of the intermetallic phases differs in the materials processed under different conditions. The lowest rotation speed led to the creation of relatively large intermetallic conglomerates. These conglomerates were gradually separated into thin rows spreading along the sample axis (extrusion direction) with the increase in both parameters of milling (Figure 2). The refinement was visible, especially in the case of 700 RPM_60 min sample, where the intermetallic phases were barely visible due to insufficient resolution of the µCT. However, their presence was previously confirmed by SEM images. In addition, a higher concentration of the intermetallic phases was observed in the middle of 300 RPM and 500 RPM samples.

The content of intermetallic phases and their properties were determined using OpenPNM software (Figure 6). Despite relatively small differences, some trends concerning intermetallic characteristics can be observed. In the case of the volume/surface ratio, the increase in time led to the formation of intermetallic particles with higher surface area, suggesting a rougher surface of the intermetallic phases. On the contrary, the samples milled for 30 min exhibited the opposite trend. Values of the aspect ratio were in good agreement with the SEM observations, where the micrographs of longitudinal sections show the alignment of the intermetallic particles along the extrusion direction. In addition, it seems that the particles’ elongation slightly increased with the increasing milling time. The median of maximal Feret diameters was in the range between 200 and 250 µm for all prepared materials, and no trend was observed concerning this characteristic. Moreover, the volume fraction of the intermetallic phases in the prepared materials was determined as well. As can be seen in Figure 6d, the volume content of the intermetallic phases did not reveal any direct connection with the milling conditions. In addition, the content of the intermetallic phases reached values up to 23 vol.%. The only exception was the 700 RPM_30 min sample (74.5 vol.%). It should be also mentioned that the resolution of the µCT was not able to distinguish all intermetallic phases in the case of the 700 RPM _60 min sample. Such a situation was caused predominantly by the low contrast between thin intermetallic rows and zinc matrix. The influence of this error will be further discussed in the Discussion section.

Pores in the material structure were characterized in the same way as the regions of the intermetallic phases. The results of the analysis are shown in Figure 7 and follow similar trend as in the case of intermetallic phases. More precisely, the pores were elongated in the direction of extrusion, and an increase in the surface area in the case of samples milled for longer times was observed (Figure 2 and Figure 7). Despite some similarities, some differences concerning the Feret diameter were also found in the results. The dependence of Feret diameters on the milling conditions is shown in Figure 7c. It is visible that both samples prepared using 300 RPM possessed large pores (215–250 µm) while the sample processed using higher rotations (500 and 700 RPM) reached 100–150 µm. As the last parameter, the volume content of the pores in comparison to the zinc matrix was determined. The content of pores did not exceed the value of 3 vol.% and the lowest value was confirmed in the case of 500 RPM samples (0.04%).

### 3.2. Mechanical Properties

**Figure 8:**
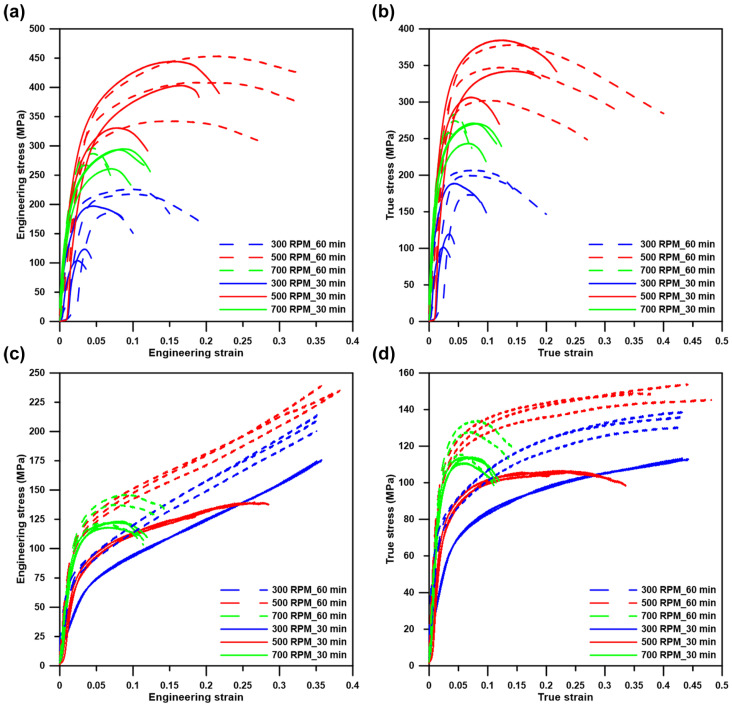
Compressive properties of as-extruded Zn-0.8Mg-0.28CaO (wt.%) alloy represented by (**a**,**c**) engineering and (**b**,**d**) true stress–strain curves measured (**a**,**b**) in the ED and (**c**,**d**) in ND.

Compressive properties were measured in both directions (ED, ND), and the curves obtained from these measurements are shown in Figure 8. It is visible in Figure 8 that the materials behaved differently in individual directions. Moreover, some trends regarding compressive behavior can be derived from the engineering and true stress–strain curves. In ED (Figure 8a,b), yield and ultimate strength increased in the following order: 300 RPM < 700 RPM < 500 RPM at both times (30 and 60 min). In addition, an increase in milling time led to an increase in CYS and UCS varying from 15–33% and from 4–29%, respectively. Additionally, it was found that the increase in milling time led to an enhancement in material plasticity. The samples milled at 700 RPM were the only exceptions, where the opposite trend was observed. On the contrary, the compressive behavior possessed different trends in ND (Figure 8c,d). The yield strength increased with the rotation speed and even with prolonged milling time in ND (Table 2), while the plasticity of the samples decreased (Figure 8c,d). Such a behavior indicates a stronger influence of the intermetallic phases and texture on the compressive performance in ND. In addition, samples loaded at ND exhibited significantly lower CYS in comparison with ED.

**Figure 9:**
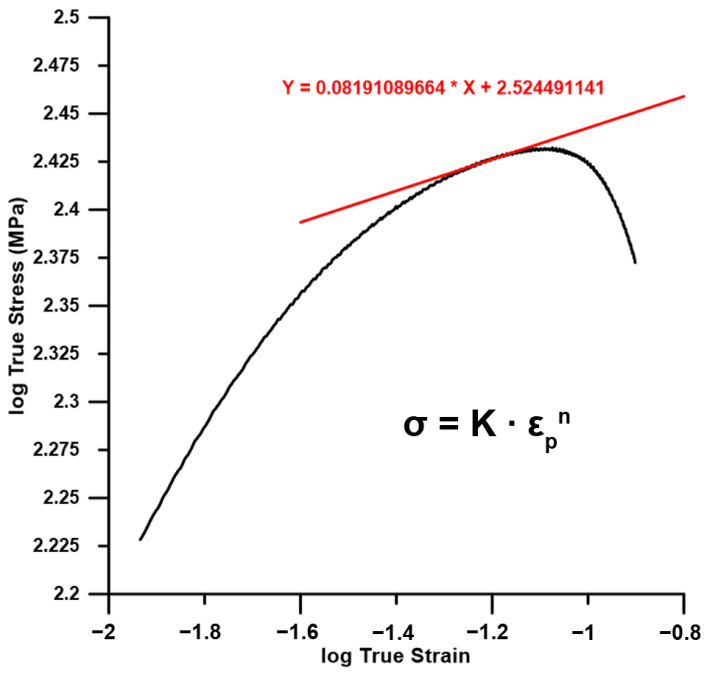
Example of the strain hardening coefficient evaluation.

The hardening parameters were also determined from true stress–strain curves (Figure 9) to reveal the possible influence of milling parameters on the resulting properties. The values of strain hardening coefficients ranged between 0.09–0.20 in ED and 0.13–0.25 for ND without any obvious connection with the milling conditions. The average values of the strengths and the hardening parameters are listed in Table 2.

## 4. Discussion

Intermetallic phases were arranged into rows and differed in the distance between them. The mutual distance between individual rows of intermetallic phases (Figure 2) was connected predominantly with the milling conditions. As was described in our previous publication concerning these alloys [39], the particles of zinc were deformed with different intensities depending on milling conditions. Generally, two important factors played a significant role, namely, the formation of the intermetallic phases on the surface of the Zn particles and the flattening of the Zn particles during the milling process. Both factors led to a direct correlation between the thickness of the processed particles and the interrow distance. Extrusion as a process has an influence on the interrow distance by the straightening of flattened particles. However, Čapek et al. [37] found that the different conditions of extrusion did not affect the interrow distances in Zn-0.8Mg-0.2Ca alloys. Despite the same extrusion condition being used in this publication, the content and distribution of intermetallic phases differ between individual extruded materials prepared from differently treated (milled) powders. This can lead to changes in the material flow through the die due to the different stiffness of the material phases and their mutual ratios. Therefore, the thickness of initial Zn particles and the content of the intermetallic phases seem to be the major parameters influencing overall inter-row distances.

**Figure 10:**
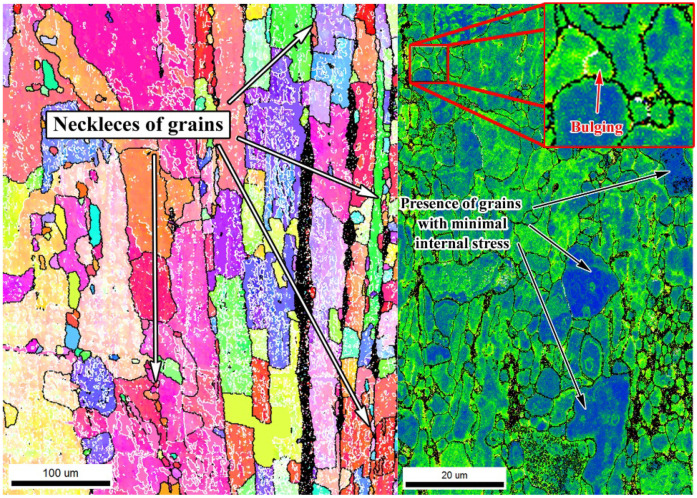
Signs of dDRX visible in IPF and Kernel average misorientation (KAM) maps of the 500 RPM_30 min sample.

It is well known that during the processing of Zn and its alloys recrystallization takes place and results in grain refinement. The recrystallization temperature of pure zinc is −12 °C [41], suggesting grain growth in the material at low temperatures. This is one of the obstacles to achieving fine-grained zinc or its alloys. However, there are many processes which can occur in the material and affect the resulting microstructure and mechanical behavior. Generally, the processes occurring during the extrusion process can be divided into three groups, containing static (SRX), dynamic (DRX), and post-dynamic (post-DRX) recrystallization. SRX can lead to a decrease in internal stresses from the milling and to a subsequent coarsening of the zinc grains. Because the process takes only 2–3 min before the extrusion (stabilizing of sample temperature in our case), the influence of SRX would be most likely very limited. During the extrusion, both types of dynamic recrystallization (DRX) took place during the processing–discontinuous (dDRX) and continuous (cDRX) dynamic recrystallization. Signs of dDRX were found and are represented by bulging, the presence of necklaces of new grains formed along grain boundaries, and the presence of stress-free grains (Figure 10) [42].

**Figure 11:**
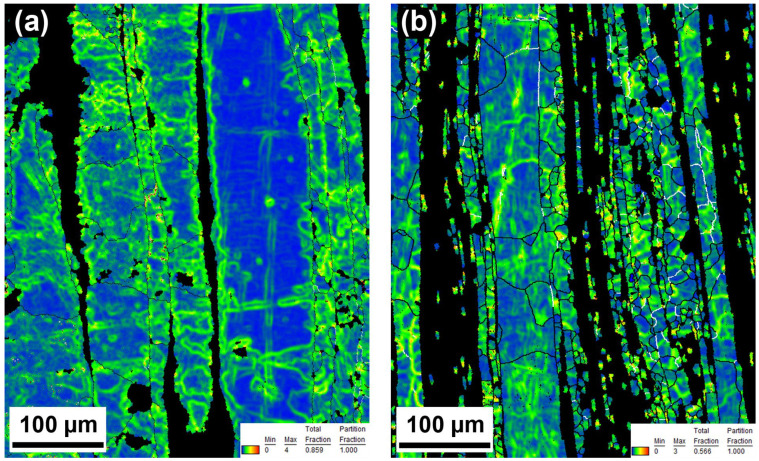
KAM of (**a**) 300 RPM_30 min and (**b**) 700 RPM_60 min samples showing the presence of inner substructures.

It is noteworthy that the necklaces of new grains could be a consequence of different processes or their combination. The formation of new grains in necklaces could be caused by the particle-stimulated nucleation process (PSN) [43]. This claim is supported by a slightly changed orientation in the closest surroundings of the intermetallic rows and relatively large conglomerates containing hard intermetallic particles (Figure 2 and Figure 5). However, there are numerous reasons to exclude the PSN process, among which the most important is the misorientation between parent–daughter grains. It was found by several authors that the orientation between dynamically recrystallized grain and the original grain reaches approximately 30° [44,45]. Based on that, we used GOS to identify non-recrystallized grains with the presence of recrystallized grains in the structure. Subsequently, mutual misorientations between these grains were measured with obtained values reaching 30°, suggesting the predominant activity of DRX rather than the PSN process (random orientation) [46]. It is known that cDRX can also lead to the formation of new grains on the grain boundaries, but the reason for that is different (compared with dDRX). The cDRX is connected with the gradual accumulation of dislocations and subsequent increase in sub-grain misorientations [42]. As evidence of cDRX, the presence of LAGB´s substructures, which gradually transform into HAGBs, can be considered (Figure 3 and Figure 4). Such substructures can be observed in Figure 11. Besides the substructures, the occurrence of cDRX could be supported by the value of the stacking fault energy of zinc (γ_SFE_). Generally, the γ_SFE_ value for zinc (140 mJ·m^−2^) is considered large, leading to a preference for cDRX. The reason is hidden in the facilitation of dynamic recovery and the inability to reach a sufficient amount of dislocation for the activity of dDRX process. On the contrary, the cDRX process can be limited by its slow kinetics and relatively low value of strain obtained by the extrusion process (ln ER = ln 10 = 2.3; ER = extrusion ratio). The aforementioned information suggests the complex character of the DRX processes and the possibility of their combination during the extrusion process. It also needs to be mentioned that we minimized the influence of thermomechanical processing by using of same conditions, such as temperature, ram speed, extrusion ratio, etc. This means that the main parameters affecting the recrystallization process were the initial state of the materials, stacking fault energy, and the presence of second-phase particles. The last process concerning the change of recrystallized microstructure, as a consequence of increased temperature, is post-DRX. However, the influence of this process was significantly decreased by quenching in cold water immediately after the extrusion. On the contrary, the process can be still active (low activity) after the quenching, especially due to the low recrystallization temperature of the zinc matrix.

**Figure 12:**
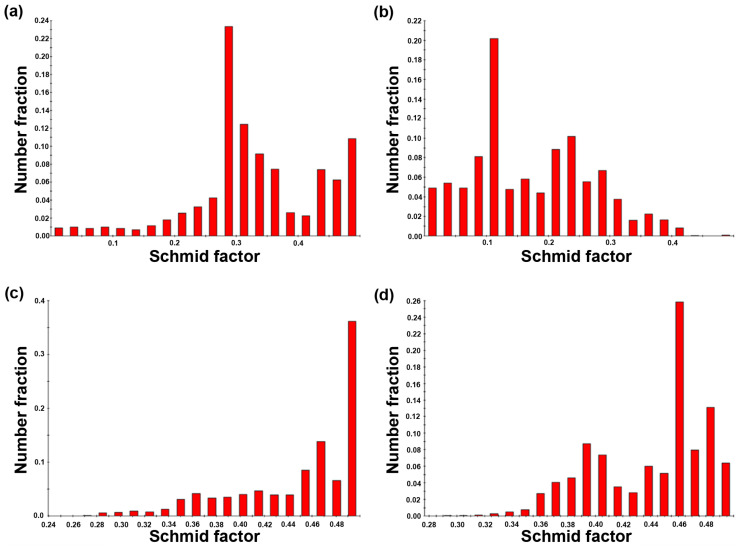
Histograms of the (**a**,**b**) basal and (**c**,**d**) pyramidal <c + a> Schmid factor evaluated in (**a**,**c**) ND and (**b**,**d**) ED.

One of the most important aims of this publication was to determine the relations between the microstructural characteristics of the materials processed under different milling conditions and the compressive properties. It is well known that the mechanical properties of the materials are affected by the grain size, texture, characteristics of second-phase particles (size, distribution, shape), and pores [47,48,49]. Based on that we observed these parameters in order to determine their effect on the resulting compressive properties. It is obvious from Figure 3, Figure 4 and Figure 5 that a finer microstructure led to higher values of CYS and UCS. On the contrary, non-linear relations could be observed in the case of 500 and 700 RPM samples. These samples possessed similar grain size values; however, the strength values did not correspond well, suggesting the influence of the other aforementioned aspects. Material texture is associated with the mechanical properties only indirectly through the preferential activity of individual deformation mechanisms. These mechanisms can be sorted by their energetic requirements to the groups representing their probable activity: 1. Basal slip, 2. Pyramidal slip, and 3. Prismatic slip [50]. This means that the activity of the basal slip is strongly expectable in the case of zinc and its alloys. In addition, the activity of twinning could be expected as well due to presence of relatively large grains [9]. However, the presence of twins was not confirmed in the material microstructure. Therefore, the activity of twinning is rather negligible. The presence of other slip systems could be also determined from the IPF figures. In the ideal case (only activity of basal slip), the basal planes are oriented parallell with the extrusion direction suggesting strong texture in the left corner of the orientation triangle in ND. Based on that, the activity of various deformation mechanisms could be revealed by the texture deviations from this corner. Deformation texture after the extrusion significantly affects mechanical properties through preferential activity of individual mechanisms during loading. To reveal these mechanisms, the values of the Schmid factor were analyzed and the Schmid histograms representing the value of the Schmid factors related to number fraction are shown in Figure 12. The obtained values of the Schmid factor confirmed the activity of basal and pyramidal <c + a> slip.

Generally, the presence of intermetallic phases is often connected with the enhancement of the mechanical properties caused by the occurrence of particles with incoherent interfaces hindering the motion of dislocations [51]. In our case, the presence of conglomerates of intermetallic particles was obvious from the SEM. These conglomerates could lead to an opposite situation (decrease in mechanical characteristics) [52]. The reason is hidden in the low cohesion between individual intermetallic particles or by the presence of pores. It can be ascribed to insufficient consolidation by the processing and, thus, to the inability to create a strong connection between the intermetallic particles. The characteristics of the intermetallic phases and pores obtained by µCT revealed some interconnections with the mechanical properties.

**Figure 13:**
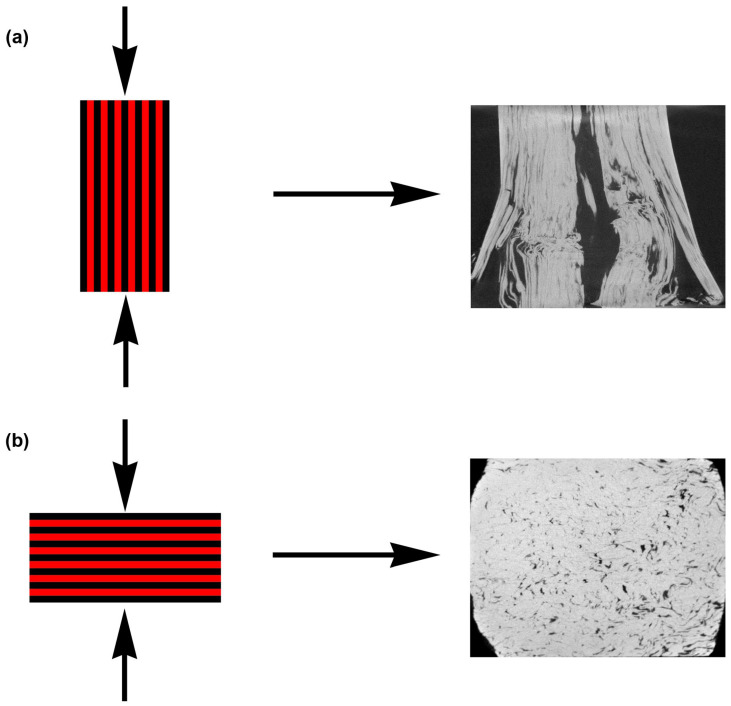
Samples loaded in the (**a**) ED and (**b**) ND with 2D scans obtained using µCT after the compressive tests.

According to SEM images (Figure 2), it is evident that the content of intermetallic phases was relatively high in the 700 RPM_60 min sample. This means that the low values obtained by µCT, shown in Figure 6d, were caused by an inability to distinguish thin rows of the intermetallic phases, which were separated by the relatively thin areas of pure zinc. Because of that, the overall contrast was small, resulting in a lower content of the intermetallic phases. This suggests that the content of intermetallic phases gradually increases in the materials with rotation speed and time. This could be ascribed to the flattening and to the subsequent wrapping of zinc particles by intermetallic phases. Together with the values of CYS, it can be concluded that the mechanical properties increased with the content of intermetallic phases in ND. In this direction, the material can be simplified as a desk-like composite, while the compressive behavior in ED is more similar to a fiber-like composite. The difference in compressive behavior between these materials is shown in Figure 13.

According to Figure 13, the influence of the individual parameters (volume/surface, aspect ratio, feret diameters, and content) on the compressive behavior in the individual directions can be deduced. In addition, the significance of these parameters would be strongly dependent on the direction of loading. In ED, higher values of feret diameters and aspect ratios can lead to the presence of larger conglomerates and pores. The pores were localized in the conglomerates of intermetallic particles rather than in the zinc matrix. Based on that, easier spreading of cracks can be observed under such conditions in ED. On the contrary, the same situation will lead to different results in ND. More precisely, the pores are compressed in this direction resulting in lower strength values but relatively good plasticity. It must be mentioned that the differences were relatively low in the selected parameters, leading to an inability to assign the influences of individual parameters reliably. In addition, the resulting behavior is most likely caused by the combination of several factors. However, the obtained alloys could be used as fiber-like composites where the synergic effect of fibers in the form of intermetallic rows and zinc matrix exhibit satisfisfactory properties in compression.

## 5. Conclusions

The Zn-0.8Mg-0.28CaO composites were successfully prepared by in situ reactions, and the mutual relations between processing conditions, microstructure, and compressive properties were determined. Obtained findings can be summarized in the following points:

(1) The distribution of the intermetallic phases in the material was affected predominantly by milling conditions.
(2) Materials underwent DRX during the extrusion process. In addition, the combination of dDRX and cDRX was confirmed by the results without the possibility to determine the prevailing ones. It was confirmed that the influence of the PSN process was rather negligible.
(3) The parameters of mechanical milling significantly affect the grain size of resulting materials after the extrusion process.
(4) Compressive properties were affected by grain size, material texture, particles of intermetallic phases, and pores.
  - Grain size had a significant effect on the compressive strengths of tested materials. With the increase in grain size, the mechanical properties decreased. However, the trend was inconsistent, suggesting the contribution of different factors to mechanical behavior.
  - Material texture pointed to activity of basal and <c + a> pyramidal slips during compression, which is in good agreement with the energetic requirements for possible slips in hcp crystal structures with a higher c/a ratio.
  - The effect of conglomerates of intermetallic particles and pores was significantly dependent on the direction of loading and subsequent progress of deformation through the material.

The mutual relations between microstructure and mechanical properties are the basics enabling the development of the material and its modification for intended applications. Based on the compressive properties, these materials seem to be suitable for further research as candidates for a fabrication of bone scaffolds; however, further research is necessary to confirm their suitability as biodegradable composites with adjustable mechanical properties.

## Figures and Tables

**Table 1:** Summarization of the milling conditions.

Sample	RPM *	Time (min)	Pause Interval/Milling Interval ** (min)	Mixture: Powder/Milling Balls Weight Ratio	Atmosphere
300 RPM_30 min	300	30	10/10	1:5	Ar
300 RPM_60 min	300	60			
500 RPM_30 min	500	30			
500 RPM_60 min	500	60			
700 RPM_30 min	700	30			
700 RPM_60 min	700	60			

* rotations per minute; ** the direction of the milling was changed after the pause interval.

**Table 2:** The overview of the compressive properties and hardening coefficients for materials processed under different milling conditions.

Direction	Sample	CYS (MPa)	UCS (MPa)	n	K (MPa)
**ED**	300 RPM_30 min	88.21 ± 12.21	138.55 ± 32.78	0.20 ± 0.10	277.15 ± 86.57
	500 RPM_30 min	152.43 ± 10.53	344.66 ± 31.97	0.09 ± 0.02	427.65 ± 44.46
	700 RPM_30 min	146.59 ± 13.96	261.82 ± 12.87	0.11 ± 0.01	357.25 ± 11.96
	300 RPM_60 min	131.69 ± 13.79	193.33 ± 14.40	0.05 ± 0.02	220.39 ± 13.71
	500 RPM_60 min	220.37 ± 14.99	343.20 ± 27.01	0.07 ± 0.01	402.21 ± 25.88
	700 RPM_60 min	170.51 ± 1.59	272.04 ± 10.45	0.10 ± 0.01	375.65 ± 4.35
**ND**	300 RPM_30 min	49.15 ± 8.68	-	0.25 ± 0.01	144.68 ± 4.07
	500 RPM_30 min	59.33 ± 3.56	106.09 ± 0.37	0.19 ± 0.03	157.80 ± 10.14
	700 RPM_30 min	76.20 ± 1.56	112.97 ± 1.51	0.17 ± 0.04	191.16 ± 20.93
	300 RPM_60 min	54.42 ± 8.67	-	0.21 ± 0.02	169.51 ± 6.95
	500 RPM_60 min	81.81 ± 1.63	-	0.13 ± 0.01	175.32 ± 4.28
	700 RPM_60 min	87.08 ± 4.64	125.82 ± 7.71	0.17 ± 0.04	205.32 ± 17.96

CYS = compressive yield strength; UCS = ultimate compressive strength.

## Data Availability

The data presented in this study are available on request from the corresponding author.
